# Delayed mite hatching in response to mechanical stimuli simulating egg predation attempts

**DOI:** 10.1038/s41598-019-50007-4

**Published:** 2019-09-16

**Authors:** Kaoru Fukuse, Shuichi Yano

**Affiliations:** 1 Saitama Agricultural Technology Research Center, Kumagaya, Saitama 360-0102 Japan; 2 Laboratory of Ecological Information, Graduate School of Agriculture, Kyoto University, Sakyo-ku, Kyoto 606-8502 Japan

**Keywords:** Evolutionary ecology, Entomology

## Abstract

Delayed or induced hatching in response to predation risk has been reported mainly in aquatic systems, where waterborne cues from predators and injured neighbouring eggs are available. Newly emerged larvae of the terrestrial predatory mite *Neoseiulus womersleyi* are vulnerable to predation by con- and heterospecific predatory mites, whereas their eggs are not. We examined whether *N. womersleyi* embryos delay hatching in response to artificial mechanical stimuli that simulates egg predation attempts. When embryos near the hatching stage were artificially stimulated every 5 min for 60 min, most stopped hatching for the duration of the 60-min period, whereas unstimulated embryos did not. Stimulated embryos resumed hatching when the treatment was stopped, and the proportion of hatched stimulated embryos caught up with that of unstimulated embryos within 120 min after stimuli stopped. Since hatching did not stop in response to changes in gravity direction, the effect of direct mechanical stimuli on the eggs was considered a proximate factor in delayed hatching. These results suggest that *N*. *womersleyi* embryos recognise immediate predation risk via mechanical stimuli, and delay hatching until the predation risk is reduced.

## Introduction

Although animals in quiescent stages cannot avoid immediate biotic risks by moving, they can avoid risks by regulating their advance toward the next developmental stages. The embryos of some aquatic animals can either induce hatching in response to egg predators^1,2^ or delay hatching in response to hatchling predators^3–5^. In such aquatic systems, water-borne chemical cues emanating from predators and/or injured conspecific eggs in the same clutch are thought to be primal proximate factors affecting induced/delayed hatching^2–5^. Although such induced/delayed hatching in response to predator cues has yet been reported in arthropods including mites, some insect embryos synchronise hatching in response to vibrations produced by their mothers^6,7^ or siblings^8^, demonstrating the potential of arthropod embryos to detect and respond to mechanical stimuli.

Terrestrial predatory mites usually feed on prey such as spider mites, whiteflies and thrips; however, they also predate juveniles of con- and heterospecific predatory mites when there is a low density of prey mites^9^. Predatory mite larvae are generally vulnerable to intraguild predation immediately after hatching, whereas eggs are not^10^ because many predatory mite species penetrate the egg chorion with difficulty^9^. The predatory mite *Neoseiulus womersleyi* oviposits solitary eggs on abaxial leaf surfaces. Although *N*. *womersleyi* adults and nymphs attempt to attack conspecific eggs (Supplemental Fig. 1), they usually fail to pierce the eggs, resulting in mechanical stimuli such as egg shaking and rolling (hereafter predation attempts), which typically last several seconds (Yano, personal observation). If the embryos can perceive and distinguish these stimuli from others, then delaying hatching for the duration of the stimuli might prevent subsequent predation after hatching.

In this study, we demonstrate for the first time that arthropod embryos delay hatching in response to mechanical stimuli simulating predation attempts by egg predators, and discuss how predatory mite embryos cope with immediate predation risks.

## Materials and Methods

### Mites

The *N*. *womersleyi* study population was collected from bushkiller plants *Cayratia japonica* (Thunb.) Gagnep. on the campus of Kyoto University in 2014 and reared on kidney bean (*Phaseolus vulgaris*) leaf disks pressed onto water-saturated cotton in Petri dishes (diameter, 90 mm; depth, 14 mm). Leaf disks were heavily infested with the two-spotted spider mite *Tetranychus urticae* Koch as prey. We placed the leaf disks in transparent plastic containers maintained at 25 °C and 50% relative humidity under a 16-h light, 8-h dark (L16:D8) photoperiod. All experiments were conducted under these conditions.

### Predation on conspecific eggs/larvae

**Figure 1:**
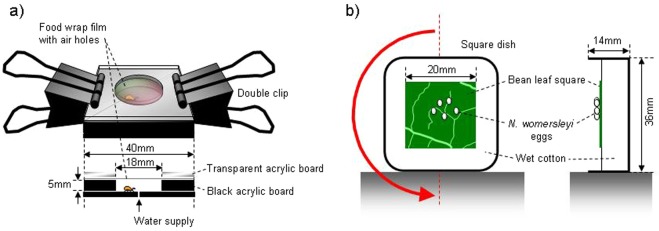
Experimental setups used to (**a**) confine *Neoseiulus womersleyi* individuals and (**b**) change the relative direction of gravity for *N. womersleyi* eggs.

To confirm that *N*. *womersleyi* eggs were less preyed upon than larvae, we randomly selected either five eggs (n = 12) or five 0-day-old larvae (n = 12) from the study population. We confined these individuals in a closed space (diameter, 18 mm; height, 5 mm, Fig. 1a) with water and air supplies according to the method of Ogawa and Osakabe^11^, together with a separately raised starved (thin) adult female *N. womersleyi*. To promote predation, we used starved females. Eggs were separately placed within the cage. We did not supply larvae with any food because they do not feed during the larval period^12^. After 24 h, the numbers of consumed eggs or larvae were recorded, and proportions of consumed eggs and larvae were compared using a Mann-Whitney *U*-test (SAS 9.22; SAS Institute Inc., Cary, NC).

### Predation attempts on conspecific eggs

To confirm egg predation attempts by conspecific adult females and describe their frequency, we controlled the hunger levels of the females by isolating them for either 1 (n = 23) or 4 (n = 21) days in 1.5-mL microtubes (Bioramo Microtube, As One, Osaka, Japan) with a water droplet. We introduced five eggs (one in the middle and four at even intervals near the periphery) on each 10-mm-diameter leaf circle. We then introduced a starved female onto the circle and observed the number and duration of predation attempts within 10 min. Attempts that lasted for more than 1 s were recorded. Numbers and total duration (s) of predation attempts were compared between females that had been starved for 1 and 4 days using a Mann-Whitney *U*-test (SAS 9.22).

### Hatching in response to mechanical stimuli

We explored whether simulated egg predation attempts could affect embryo hatching as follows. To obtain ready-to-hatch embryos, we prepared cohorts of *N. womersleyi* eggs. This process was necessary due to the extreme difficulty of screening embryos just before hatching without careful examination from all directions (i.e., stimulation). We prepared two egg cohorts (n = 62, 61) by collecting eggs oviposited by 240 adult female *N*. *womersleyi* within 3 h. We transferred these to one half of one side of each new leaf disk. After 2 days, when 30% of the embryos in each cohort had hatched (i.e., remaining embryos were thought to be nearly hatching), we transferred half of the remaining eggs to symmetrical positions (i.e., the other half) on the leaf disks using a fine brush and manipulated them as described below.

Due to the low frequency of egg predation attempts by starved adult females (less than once per egg within 10 min), we artificially simulated mechanical stimuli that occur during egg predation attempts instead of using actual predators. To simulate immediate predation risk, we stimulated individual eggs by rolling them ca. 90° around their longer diameter with a fine brush every 5 min for 60 min (stimulated eggs, n = 35). We could not stimulate eggs more frequently because each simulated predation attempt involving all 35 eggs required nearly 5 min to complete. The eggs remaining on the other side of the leaf served as a control group (n = 39). We recorded the number of hatched embryos every 15 min until 120 min after the stimulation treatment when the proportion of hatched embryos caught up with that of unstimulated embryos, and removed hatched larvae from among both stimulated and control eggs. Data from two cohorts were pooled for analysis. We compared the proportion of hatched embryos between the stimulated and control eggs at the end of the treatment period (60 min) and again at the end of the experiment (180 min) using Fisher’s exact test (SAS 9.22).

### Hatching in response to changes in gravity direction

We explored whether relative changes in the direction of gravity can affect embryo hatching. This experiment was intended to simulate physical stimuli other than predation attempts. We prepared eggs near the hatching stage in the manner described above and transferred them onto a 20 × 20-mm leaf square pressed onto water-saturated cotton in a square dish (36 × 36 mm; depth, 14 mm). The dish was placed on its side to keep the leaf surface perpendicular; the eggs did not fall off because their surfaces are sticky (Fig. 1b). To change the relative gravity direction, the dish was turned upside down every 5 min for 60 min (gravity inversion, n = 72). Eggs on a fixed perpendicular dish served as a control group (n = 76). We observed the number of hatched embryos on perpendicular leaf surfaces every 15 min using a magnifying lens, and compared the proportion of hatched embryos between treatment and control eggs at the end of the experiment (60 min) using Fisher’s exact test (SAS 9.22).

### Ethical approval

This article does not contain any studies with human participants or animals performed by any of the authors.

## Results

### Predation on conspecific eggs/larvae

**Figure 2:**
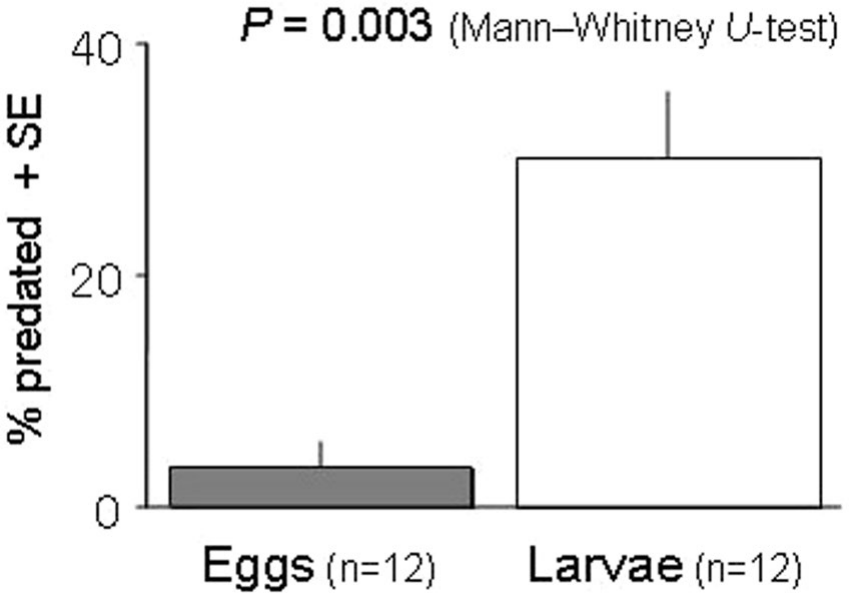
Predation on conspecific eggs/larvae. The proportion of *N*. *womersleyi* offspring preyed upon by a conspecific adult female differed significantly between eggs and larvae (*P* = 0.003, Mann–Whitney *U*-test).

The mean ± SE proportion of *N*. *womersleyi* eggs preyed upon by a conspecific adult female was 3.33 ± 2.24% (n = 12), whereas that of larvae was 31.30 ± 6.45% (n = 12). These proportions differed significantly (*P* = 0.003, Mann–Whitney *U*-test, Fig. 2).

### Predation attempts on conspecific eggs

**Figure 3:**
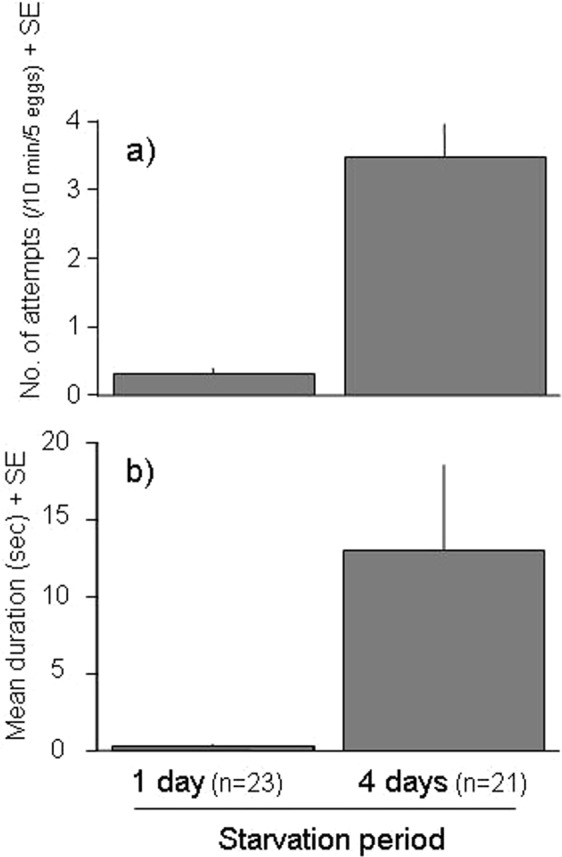
Predation attempts on conspecific eggs. The number of predation attempts by a starved adult female on five conspecific eggs within 10 minutes differed significantly between females that had been starved for 1 and 4 days (*P* < 0.0001, Mann–Whitney *U*-test, Fig. 3a). The total duration (seconds) of predation attempts also differed significantly (*P* < 0.0001, Mann–Whitney *U-*test, Fig. 3b).

The number of predation attempts on five eggs within 10 min differed significantly between females that had been starved for 1 and 4 days (*P* < 0.0001, Mann–Whitney *U*-test, Fig. 3a). The total duration (s) of predation attempts within 10 min also differed significantly between these groups (*P* < 0.0001, Mann–Whitney *U-*test, Fig. 3b). The maximum duration of predation attempts was 114 s, by a female starved for 4 days.

### Hatching in response to mechanical stimuli

**Figure 4:**
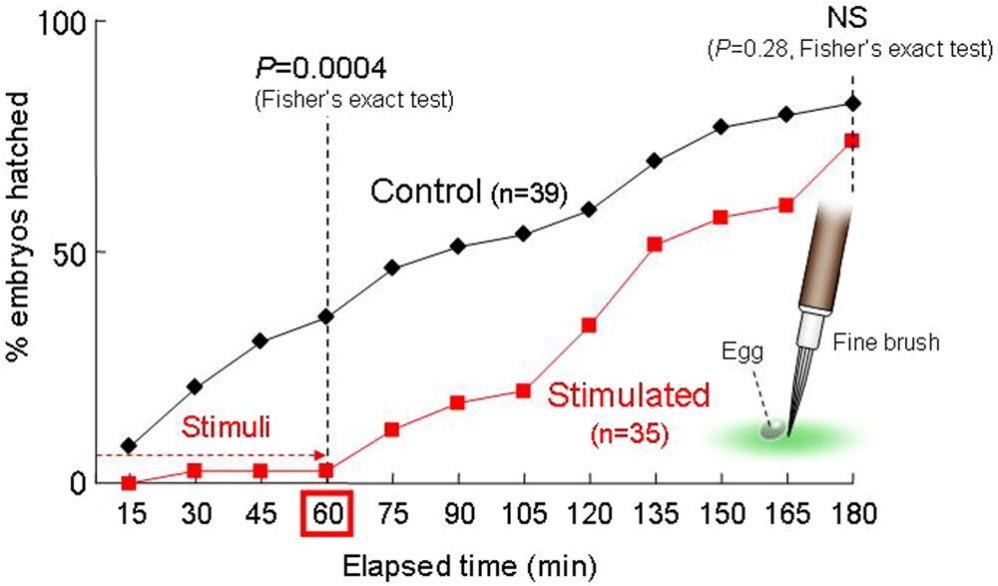
Hatching in response to mechanical stimuli. The proportions of hatched embryos differed significantly between stimulated and control eggs (*P* = 0.0004, Fisher’s exact test) at the end of the treatment period (60 min) but not after the subsequent 120 min (180 mm) (*P* = 0.28, Fisher’s exact test).

Most stimulated embryos did not hatch during the treatment period (i.e., initial 60 min), and the proportions of hatched embryos at the end of the treatment period (60 min) differed significantly between stimulated and control eggs (*P* = 0.0004, Fisher’s exact test, Fig. 4). However, stimulated embryos resumed hatching after the treatment was stopped, and the proportion of hatched embryos caught up with that of unstimulated embryos in the subsequent 120 min (*P* = 0.28, Fisher’s exact test).

### Hatching in response to changes in gravity direction

**Figure 5:**
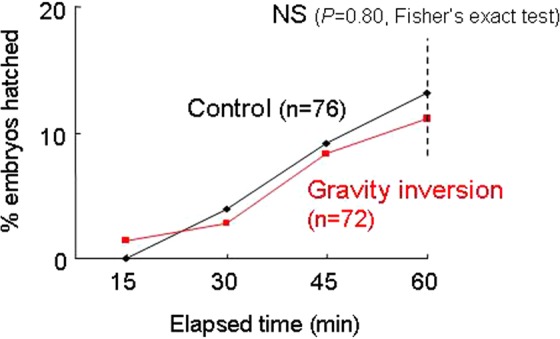
Hatching in response to changes in the relative direction of gravity. The proportions of hatched embryos at the end of the gravity inversion treatment (60 min) did not differ significantly between gravity inversion and control eggs (*P* = 0.80, Fisher’s exact test).

The proportions of hatched embryos at the end of the gravity inversion experiment (60 min) did not differ significantly between the treatment and control eggs (*P* = 0.80, Fisher’s exact test, Fig. 5).

## Discussion

We demonstrated that nearly hatching embryos of predatory mites delayed hatching in response to periodic mechanical stimuli that simulated predation attempts by con- or heterospecific predatory mites. Stimulated embryos resumed hatching after treatment was stopped, clearly indicating that the lower hatching rate observed during treatment was not due to lethal damage caused by the treatment. We also demonstrated that the embryos did not stop hatching in response to changes in gravity direction, which can be caused by natural disturbances such as the wind shaking eggs on leaves. Such disturbances change the direction that gravity acts on the eggs without touching the eggs. Therefore, embryo hatching may cease in response to direct mechanical stimuli or changes in the egg part contacting the leaf surface as a result of such stimuli, likely as an adaptation against predation attempts by predatory mites. Egg predation attempts by conspecifics did occur under experimental conditions, but their frequency seemed to depend on predator conditions. We also demonstrated that eggs are safer than larvae in the presence of conspecific adults. As predatory mite eggs are generally better protected against such predators than are larvae just after hatching^9,10^, eggs under immediate predation risks remain safer if the egg stage is prolonged. We therefore hypothesised that predatory mite embryos delay their hatching in response to an immediate predation risk until the risk is reduced. Because predation attempts on *N*. *womersleyi* eggs by conspecifics lasted for a few minutes at most, the ability of embryos to delay hatching by at least 60 min seems sufficient to mitigate predation risk. Interestingly, stimulated embryos did not hatch immediately after simulated stimuli were stopped, but seemed to hatch gradually over time. This cautious embryo hatching after the stimuli ended may reflect an ‘arms race’ between eggs and predatory mites that might not move away, but rather wait near the eggs for larvae to hatch^9,13^.

We demonstrated that mechanical stimuli alone can delay hatching in predatory mite embryos. Induced hatching in response to mechanical vibration has been reported in egg masses of a wide range of animals including reptiles^14^ and insects^6–8^; however, this study is the first report of delayed hatching in response to predator-induced mechanical stimuli. Although it is possible that contact and/or airborne chemical cues from predators may also affect the hatching timing of predatory mite embryos, we speculate that these cues are less important for the following reasons. First, the mites do not oviposit in clusters; we preliminarily observed that none of 32 eggs oviposited on a leaf by 15 females within 24 h were in contact with one another. Therefore, chemical cues from predators attacking nearby conspecific eggs would likely be rare. Second, unlike water-borne chemical cues that affect embryo hatching in aquatic animals^2–5^, airborne chemical cues from a distance appear barely detectable by some predatory mites^15,16^. In contrast, direct mechanical stimuli on eggs, which are generally well defended against predators, may be primary cues of immediate predation risks, as only eggs under attack receive these stimuli. In addition, embryos may respond to non-specific mechanical stimuli because they are typically attacked by both con- and heterospecific predatory mites^17^, which may possess different forms of contact and/or volatile chemicals.

We hypothesise that delayed embryo hatching in response to predator-induced mechanical stimuli may be widespread in solitary eggs that are robustly defended against predation and in systems in which chemical predation cues are difficult to detect. This hypothesis should be addressed in future studies, especially for arthropods inhabiting terrestrial systems.

## Supplementary information

Supplementary Figure 1

Supplementary dataset 1

Supplementary dataset 2

Supplementary dataset 3

Supplementary dataset 4

## Data Availability

All data can be found in the Supplementary Material.
