# Supplementary material for: Delayed mite hatching in response to mechanical stimuli simulating egg predation attempts
Source: Sci Rep. 2019 Sep 16;9:13395. doi: 10.1038/s41598-019-50007-4 (PMC6746699; doi:10.1038/s41598-019-50007-4)
Supplement: Supplementary file 1 — Supplementary Figure 1 [file 41598_2019_50007_MOESM1_ESM.pdf]

Title: Delayed mite hatching in response to mechanical stimuli simulating egg predation attempts

Author list: Kaoru Fukuse, Shuichi Yano

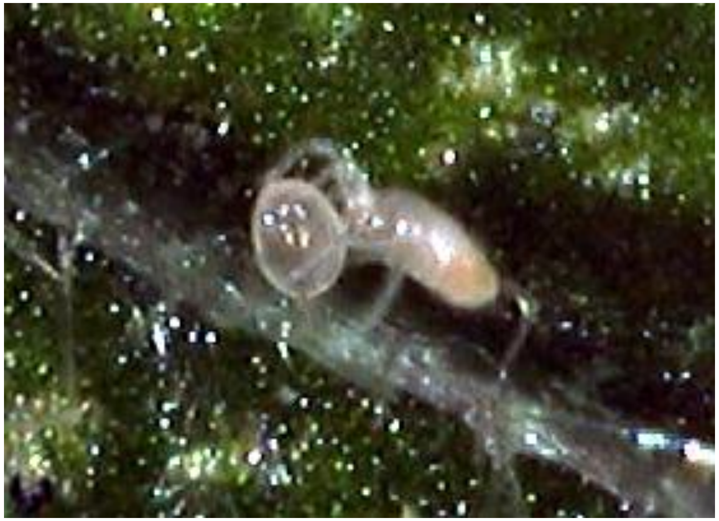

Supplementary Figure 1
*Neoseiulus womersleyi* deutonymph attempting to attack a conspecific egg.
